# Dysfunctional Muscle and Liver Glycogen Metabolism in *mdx* Dystrophic Mice

**DOI:** 10.1371/journal.pone.0091514

**Published:** 2014-03-13

**Authors:** David I. Stapleton, Xianzhong Lau, Marcelo Flores, Jennifer Trieu, Stefan M. Gehrig, Annabel Chee, Timur Naim, Gordon S. Lynch, René Koopman

**Affiliations:** Department of Physiology, The University of Melbourne, Melbourne, Victoria, Australia; Goethe University, Germany

## Abstract

**Background:**

Duchenne muscular dystrophy (DMD) is a severe, genetic muscle wasting disorder characterised by progressive muscle weakness. DMD is caused by mutations in the dystrophin (*dmd*) gene resulting in very low levels or a complete absence of the dystrophin protein, a key structural element of muscle fibres which is responsible for the proper transmission of force. In the absence of dystrophin, muscle fibres become damaged easily during contraction resulting in their degeneration. DMD patients and *mdx* mice (an animal model of DMD) exhibit altered metabolic disturbances that cannot be attributed to the loss of dystrophin directly. We tested the hypothesis that glycogen metabolism is defective in *mdx* dystrophic mice.

**Results:**

Dystrophic *mdx* mice had increased skeletal muscle glycogen (79%, (P<0.01)). Skeletal muscle glycogen synthesis is initiated by glycogenin, the expression of which was increased by 50% in *mdx* mice (P<0.0001). Glycogen synthase activity was 12% higher (P<0.05) but glycogen branching enzyme activity was 70% lower (P<0.01) in *mdx* compared with wild-type mice. The rate-limiting enzyme for glycogen breakdown, glycogen phosphorylase, had 62% lower activity (P<0.01) in *mdx* mice resulting from a 24% reduction in PKA activity (P<0.01). In *mdx* mice glycogen debranching enzyme expression was 50% higher (P<0.001) together with starch-binding domain protein 1 (219% higher; P<0.01). In addition, *mdx* mice were glucose intolerant (P<0.01) and had 30% less liver glycogen (P<0.05) compared with control mice. Subsequent analysis of the enzymes dysregulated in skeletal muscle glycogen metabolism in *mdx* mice identified reduced glycogenin protein expression (46% less; P<0.05) as a possible cause of this phenotype.

**Conclusion:**

We identified that *mdx* mice were glucose intolerant, and had increased skeletal muscle glycogen but reduced amounts of liver glycogen.

## Introduction

Duchenne muscular dystrophy (DMD) is a severe, genetic muscle wasting disorder characterised by progressive muscle weakness that culminates in respiratory failure and death, usually in the second to third decade of life. The disease affects approximately 1∶3500 live male births worldwide with affected boys usually wheelchair bound by their early teens and experiencing a severely reduced quality of life. DMD is caused by mutations in the dystrophin (*dmd*) gene resulting in very low levels or the complete absence of the dystrophin protein, a key structural element of muscle fibres involved in force transmission. In the absence of dystrophin, muscle fibres are easily damaged during contraction resulting in their breakdown. As skeletal muscle has an inherent capacity to regenerate through the activation, proliferation, and differentiation of satellite cells, dystrophic muscle are characterised by ongoing cycles of degeneration and regeneration and an environment of low grade inflammation. As a consequence, the regenerative capacity of dystrophic muscle is compromised, resulting in the deposition of adipose and fibrotic tissue.

DMD patients, especially those that are wheelchair-bound, exhibit mild glucose intolerance and hyperinsulinemia [1]. Patients and *mdx* mice show metabolic disturbances that cannot be attributed solely and directly to the loss of dystrophin, and may reflect loss of function of other proteins in the dystrophin-associated glycoprotein complex (DGC) or from defects in glycolysis [2]. It has been reported that carbohydrate metabolism in skeletal muscles of dystrophic mice is altered considerably with reports of elevated muscle glycogen content [3], increased rates of glycogen synthesis (as measured by incorporation of ^14^C into glycogen) and a decreased ability to produce lactate from glycogen [4], consistent with reductions in glycogen phosphorylase activity [4]. Glycogen is a branched polymer of glucose comprised of α-1,4-glycosidic bonds with α-1,6-glycosidic linkages at branch points [5]. Glycogen synthesis is initiated by the autoglucosylation of glycogenin and elongated by the activities of glycogen synthase (GS, α-1,4-glycosidic links) and glycogen branching enzyme (GBE, α1,6-glycosidic shorter branches). GBE excises a segment of existing oligosaccharide on glycogen by cleaving an α-1,4-glycosidic linkage, and reforming an α-1,6-glycosidic linkage [6], [7]. Glucose is mobilized from glycogen by the concerted action of glycogen phosphorylase (GP) and glycogen debranching enzyme (GDE) acting in reverse to GS and GBE [5]. Glycogen granules consist of several tiers of glucose moieties, with 30–45% of the glucose being present in the outer tiers and thereby branched with α-1,4-glycosidic links and requiring GP for utilization. Muscle glycogen is important for providing glucose as a source of ATP for energy-requiring events like muscle contraction and calcium handling [8].

Based on this information regarding glycogen metabolism and the reports of mild glucose intolerance in DMD patients, we tested the hypothesis that altered glycogen metabolism in skeletal muscles of *mdx* dystrophic mice results in systemic metabolic alterations that affect liver glycogen metabolism and glucose tolerance.

## Materials and Methods

### Animals

All experiments were approved by the Animal Ethics Committee of The University of Melbourne and conducted in accordance with the Australian code of practice for the care and use of animals for scientific purposes as stipulated by the National Health and Medical Research Council (Australia). Male wild type C57BL/10ScSn (BL10) and C57BL/10ScSn-*dmd^mdx^*/J (*mdx*) dystrophic mice (16 weeks old; *n = *8 per group; 32 total) were obtained from the Animal Resources Centre (Canning Vale, WA, Australia) and housed in the Biological Research Facility at The University of Melbourne under a 12-hour light/dark cycle. All mice were housed in boxes of four. Animal numbers were based on previous work in our lab [9]. The animals were provided access to drinking water and standard chow *ad libitum* and monitored daily prior to the experiments.

### Whole-body Functional Assessments

Two days before endpoint dissections, whole body strength and mobility and coordination were assessed by means of a grip strength meter (Columbus Instruments, Columbus, OH) and rotarod performance (Rotamex-5, Columbus Instruments) as described previously [10]. Both assessments were performed in an alternating order between groups, with one full box of mice tested at a time. The observations that both grip strength and rotarod performance were 30% lower in *mdx* compared with C57BL/10 mice (data not shown) confirmed muscle weakness in this mouse model of muscular dystrophy.

### Glucose Tolerance Testing

Glucose tolerance tests were performed on wild type C57BL/10 and *mdx* mice (n = 8 per group). Following an overnight fast, a basal blood sample was taken from the tail vein (23 G needle) and analysed for glucose concentration using a handheld glucometer (Accu-Chek Performa, Roche Diagnostics Australia, Castle Hill, NSW, Australia). Mice then received an intraperitoneal (*i.p.*) injection of glucose solution (1 g/kg body mass). At 15, 30, 60, 90 and 120 min after the injection of the glucose solution, a blood sample was collected from the tail vein (23 G needle) and analysed for glucose concentration.

### Tissue Collection

Ad libitum fed mice (8 C57BL/10 and 8* mdx,* weighing 30.9±0.5 and 34.7±0.4 gram respectively, *P*<0.0001) were anesthetized at 9 AM with sodium pentobarbitone (Nembutal, 60 mg/kg, Sigma-Aldrich Co., Castle Hill, NSW, Australia) via *i.p*. injection in our laboratory, such that they were unresponsive to tactile stimuli. Blood was collected from the abdominal aorta in EDTA-containing tubes and centrifuged at 1000 *g* and 4°C for 10 min. Plasma was frozen in liquid nitrogen and stored at –80°C. After careful excision of the tibialis anterior (TA), plantaris (PLAN), gastrocnemius (GAST), soleus (SOL) and quadriceps (QUAD) muscles, the liver, and epididymal fat, mice were killed by cardiac excision while still deeply anesthetized. Tissues were blotted on filter paper and weighed on an analytical balance. The right TA muscle was mounted in embedding medium and frozen in thawing isopentane, while the other muscles were frozen directly in liquid nitrogen and stored at −80°C for subsequent analyses.

### Tissue Analyses

#### Glycogen and glycogen metabolic enzyme analysis

Liver and muscle tissues were homogenized and assessed for glycogen amount [11], glycogen synthase [12], glycogen phosphorylase [13] and glycogen branching enzyme activities [14]. In addition, fibre type-specific muscle glycogen content was determined by periodic acid-Schiff (PAS) staining as described previously [15].

#### Western blotting

For determination of glycogenin protein expression, 0.4 µg of PMSF-treated α-amylase (Sigma-Aldrich, MO, USA) was added to 50 µg tissue homogenate and incubated at room temperature (RT) for 20 minutes, prior to SDS-PAGE and subsequent Western blot analysis. Rabbit anti-human glycogenin primary antibodies [11] were incubated overnight at 4°C and detected by swine anti-rabbit HRP (Dako, Glostrup, Denmark) and subsequent ECL [11]. Glyceraldehyde-3-phosphate dehydrogenase (GAPDH) was used as a loading control. Antibodies to glycogen-debranching enzyme and starch-binding domain protein 1 (STBD1) were used as described previously [16]. Representative blots are provided in Supplemental Figure 1.

**Figure 1:**
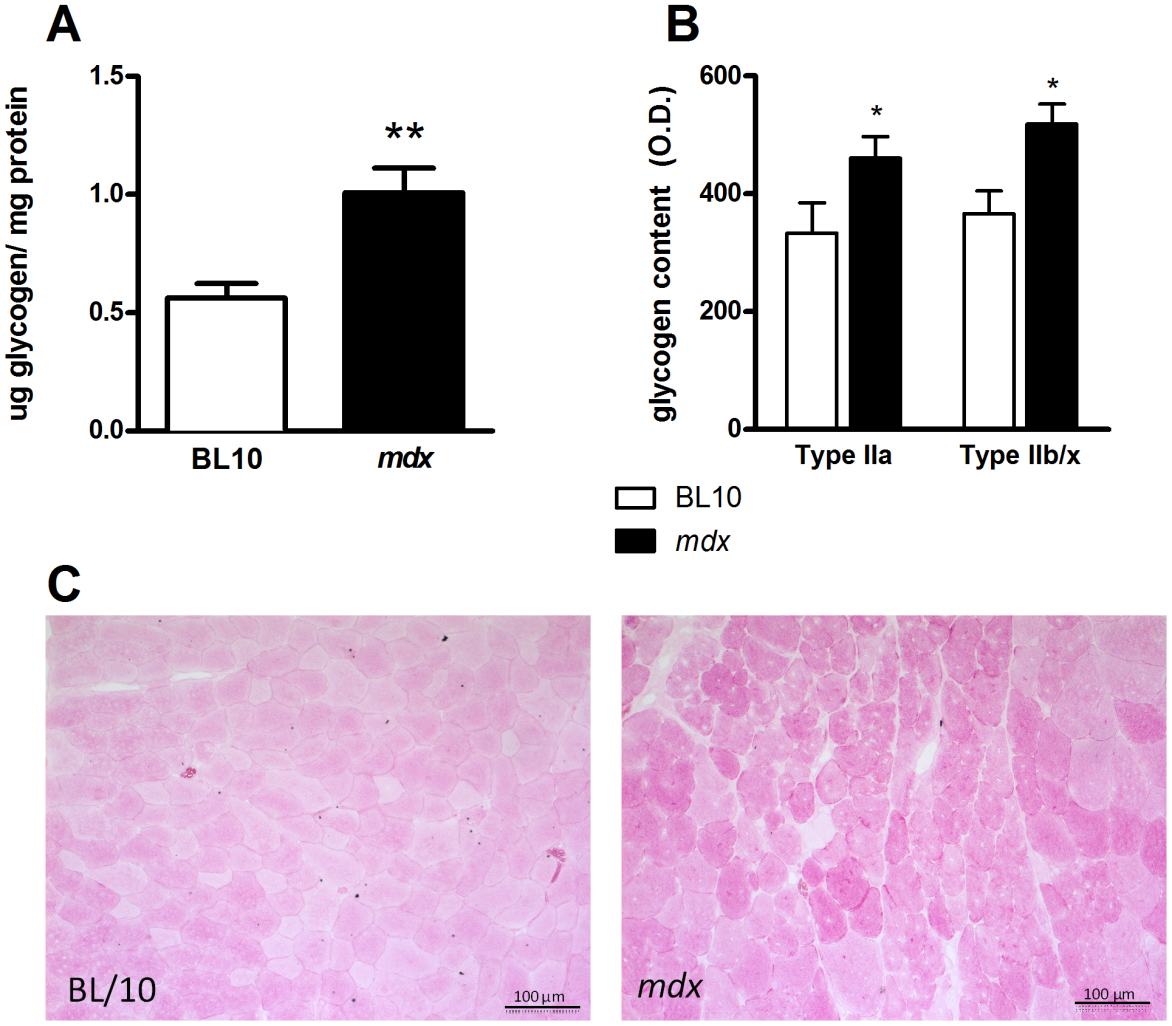
Skeletal muscle glycogen content is increased in *mdx* mice. Means (± SEM) for (**A**) muscle glycogen concentration, determined using biochemical assays; (**B**) muscle fibre type-specific glycogen content, expressed as average optical density per fibre; (**C**) representative images of period acid-Schiff (PAS) stained muscle cross-sections from BL/10 wild type and *mdx* mice; n = 8; **P<0.01, *P<0.05, compared with skeletal muscle from BL/10 wild type mice.

#### Real-Time RT-PCR

Real-Time RT-PCR was performed as described previously [10]. Total RNA was extracted from 10–20 mg of quadriceps muscle using a commercially available kit, according to the manufacturer’s instructions (PureLink RNA Mini Kit, Invitrogen, Carlsbad, CA; USA). RNA was transcribed into cDNA using the Invitrogen SuperScript VILO cDNA Synthesis Kit, and the resulting cDNA was stored at −20°C for subsequent analysis. Real-Time PCR was performed using the Bio-Rad iCycler Thermal Cycler. The forward and reverse primer sequences used were: Gyg, 5′-GGTGGCCTGACTGTTTCAAT-3′and 5′-CAAATGGCAGTTTTGTG-3′; and Stbd1, 5′-TGCAGAATGTTGGAGCAGAC-3′ and 5′-CTTTGATTTCTTGCCGCTTC-3′. Measurements included a no-template control as well as an RT (reverse transcription) negative control. The content of single stranded DNA (ssDNA) in each sample was determined using the Quanti-iT OliGreen ssDNA Assay Kit (Molecular Probes, Eugene, OR; USA), as described previously. Gene expression was quantified by normalizing to the cDNA content of each sample and expressed as arbitrary units (AU).

### Statistics

Data were expressed as mean ± SEM. Unpaired t tests were applied to determine differences between wild-type and *mdx* mice. The level of statistical significance was set at *P*<0.05 for all comparisons. All calculations were performed using GraphPad Prism 5 (GraphPad Software Inc., La Jolla, CA, USA).

## Results

### Increased Glycogen Content in Skeletal Muscles of mdx Mice

Previous studies have reported increased glycogen [17] and decreased glycogenolysis [4], [18] in the muscles of boys with DMD. We therefore investigated glycogen metabolism in *mdx* mice, a murine model for DMD [19]. First we measured the glycogen concentration in skeletal muscles from *mdx* mice and age matched C57BL/10 control mice in both the fed and fasted state. Glycogen content was increased in quadriceps and TA muscles of *mdx* mice as determined from biochemical analysis (Fig. 1A) in a fibre-dependent manner with glycogen content increased in both slow- and fast-twitch muscle fibres (Fig. 1B). PAS staining confirmed the increase in muscle glycogen in *mdx* mice (Fig. 1C). There was no difference in skeletal muscle glycogen content between control and *mdx* mice in the fasted state (data not shown), although it was evident that fasting reduced muscle glycogen content. Given that *mdx* mice displayed increased muscle glycogen content in the fed state we examined the molecular basis for impaired glycogen metabolism under these conditions.

### Increased Glycogen Synthesis in Skeletal Muscles of mdx Mice

**Figure 2:**
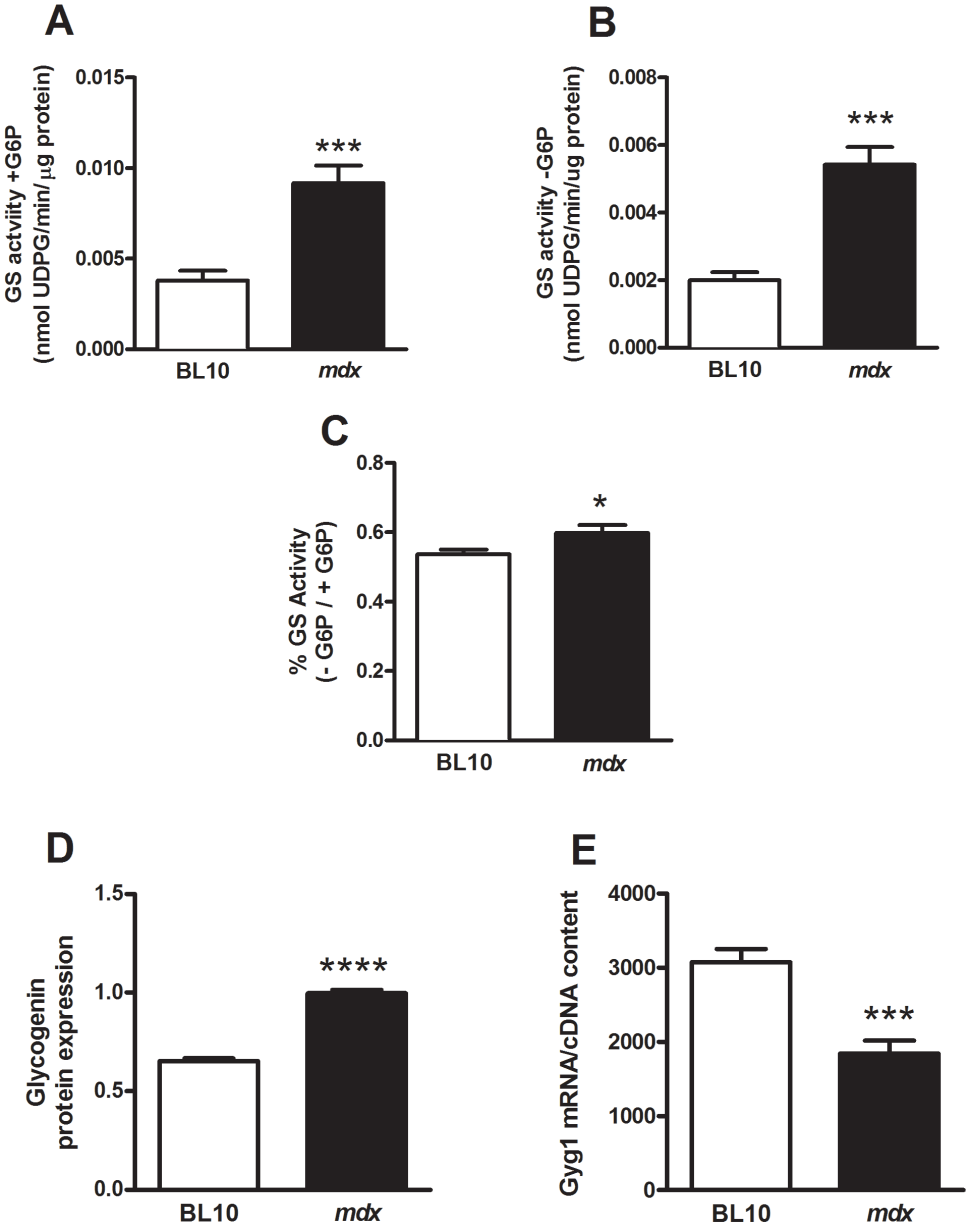
Increased glycogen synthesis contributes to the increased muscle glycogen concentration in *mdx* mice. Means (± SEM) for the ratio of skeletal muscle glycogen synthase activity in the absence (**A**) and presence (**B**) of glucose-6-phosphate (G6P) and the ratio between the two (**C**). Thus, glycogen synthase activity is higher in *mdx* mice contributing to increased glycogen in skeletal muscle. Glycogenin protein (**D**) and mRNA (**E**) expression was detected by immunoblot following α-amylase incubation, and Real-Time RT PCR, respectively, as described in the Materials and Methods. n = 8; ****P<0.0001, ***P<0.001 and *P<0.05, compared with skeletal muscle from BL/10 wild type mice.

To determine whether the increased glycogen content was due to an imbalance between synthesis and degradation we first measured the rate-limiting enzyme important for glycogen synthesis; glycogen synthase (GS). In both the absence (Fig. 2A) and presence (Fig. 2B) of the allosteric activator glucose-6-phosphate (G6P), that drives conversion of GS to its fully active state, muscles from *mdx* mice displayed increased GS activity compared with muscles from control mice (Fig. 2A/B) leading to an increase in the proportion of active/inactive conformations as shown by the –G6P/+G6P (I/T) ratio (Fig. 2C). Additionally, glycogenin protein expression was measured to determine if it also contributed to the presence of increased glycogen together with increased GS activities. Muscle lysates were incubated with α-amylase, an enzyme that digests the glycogen particle and exposes glycogenin at the glycogen core [20], and assessed for glycogenin expression via Western blotting. Protein expression for glycogenin was two-fold higher in skeletal muscles of *mdx* compared with wild-type mice (Fig. 2D). Muscle lysates that were not pre-treated with α-amylase did not exhibit immunoreactivity for glycogenin (data not shown). However mRNA levels for glycogenin were two-fold lower in skeletal muscles from *mdx* mice (Fig. 2E).

### Decreased Glycogen Breakdown in Skeletal Muscles of mdx Mice

**Figure 3:**
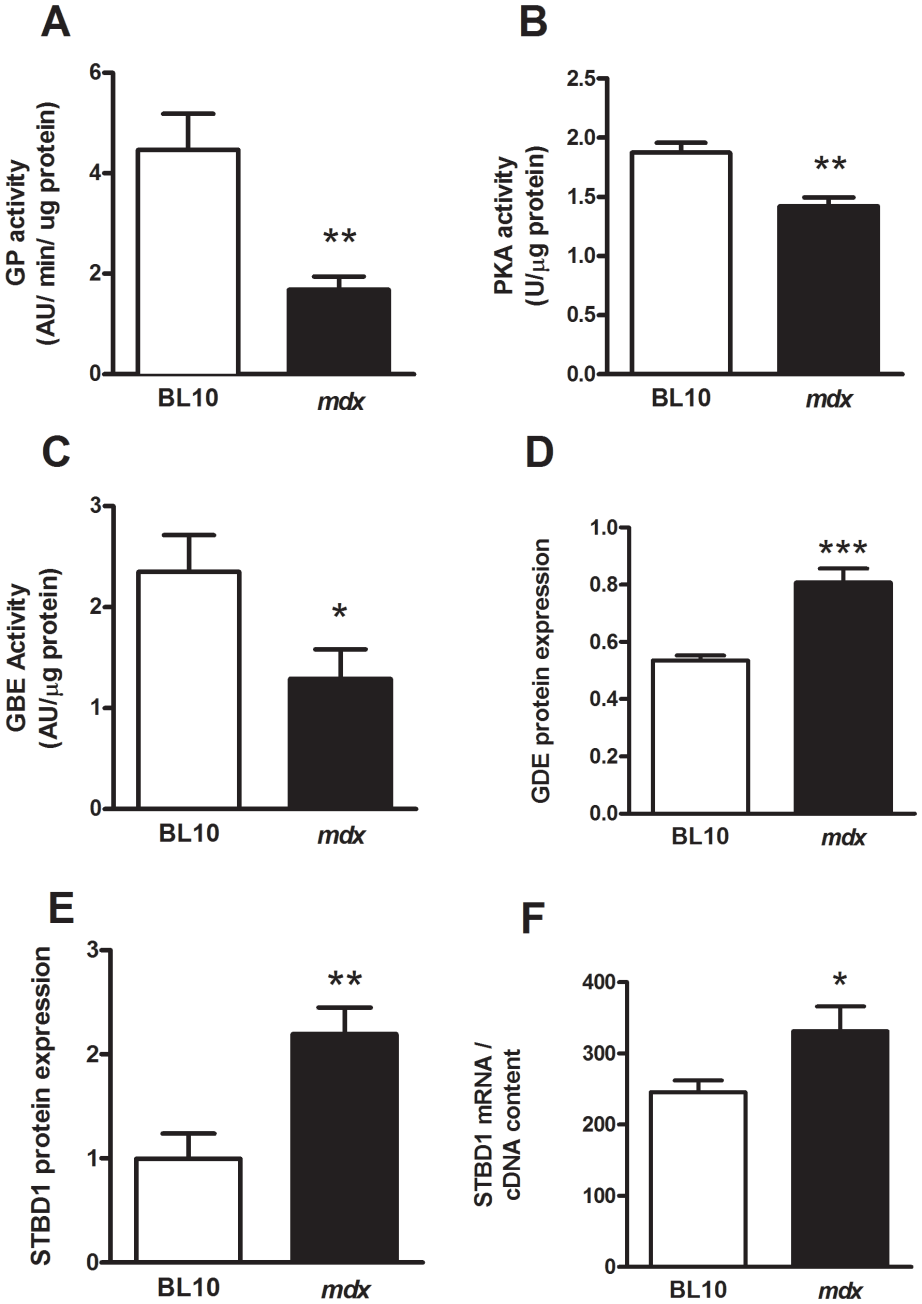
Altered glycogen metabolism contributes to the increased muscle glycogen concentration in *mdx* mice. Means (± SEM) for glycogen phosphorylase (**A**) and PKA activity (**B**) in skeletal muscles from BL10 wild type and *mdx*. n = 8; **P = 0.01 compared with skeletal muscle from BL/10 wild type mice. Means (± SEM) for glycogen branching enzyme (GBE, **C**) and glycogen debranching enzyme (GDE, **D**) activity in skeletal muscle from BL10 wild type and *mdx* mice. Protein expression was detected by immunoblot and its relative level of expression normalized against GAPDH. Data represented as ± SEM, n = 8; ***P = 0.001 compared with skeletal muscle from BL/10 wild type mice. Means (± SEM) for starch-binding domain containing protein 1 (STBD1) protein (**E**) and mRNA (**F**) expression relative to GAPDH or cDNA content, respectively. **P<0.01 compared with skeletal muscle from BL/10 wild type mice.

To determine whether the increased glycogen in muscles from *mdx* mice could also be attributed to attenuation in glycogen breakdown we measured glycogen phosphorylase (GP) activity and found that GP activity decreased by 50% in skeletal muscles of *mdx* mice (Fig. 3A). GP is primarily regulated by phosphorylation on Ser-14 by glycogen phosphorylase kinase that in turn is activated by cAMP-dependent protein kinase (PKA). Given the decrease in GP activity we hypothesized that PKA activity would also be decreased in muscles of *mdx* mice compared with control and this was confirmed based on the 20% significant decrease in activity (Fig. 3B).

### Evidence of Altered Glycogen Particle Structure in Skeletal Muscles of mdx Mice

Glycogen is a branched polymer of glucose requiring the glycogen branching enzyme (GBE) to add branched points within each linear chain created by GS and the debranching enzyme (GDE) to remove these during glycogen degradation. We found a 50% decrease in GBE activity in skeletal muscle lysates compared with control mice (Fig. 3C). Determination of GDE activity is complicated by the presence of two catalytic sites within this enzyme and therefore we measured GDE protein expression. As shown in Figure 3D we found a two-fold increase in GDE protein expression in muscle lysates from *mdx* mice. A recently described pathway for the degradation of glycogen, known as glycophagy [21], has been proposed to remove incorrectly branched glycogen by the interaction of the glycogen-associated protein starch-binding domain protein 1 (STBD1) with glycogen [22]. In confirmation of a potentially altered glycogen particle structure in the muscles from *mdx* mice we found a two-fold increase in both STBD1 protein expression and mRNA levels (Fig. 3E/F) suggesting that muscles from *mdx* mice were utilizing an alternative pathway to degrade glycogen.

### Impaired Glucose Tolerance in mdx Mice: Evidence of Decreased Liver Glycogen Content due to Decreased Glycogenin Expression

**Figure 4:**
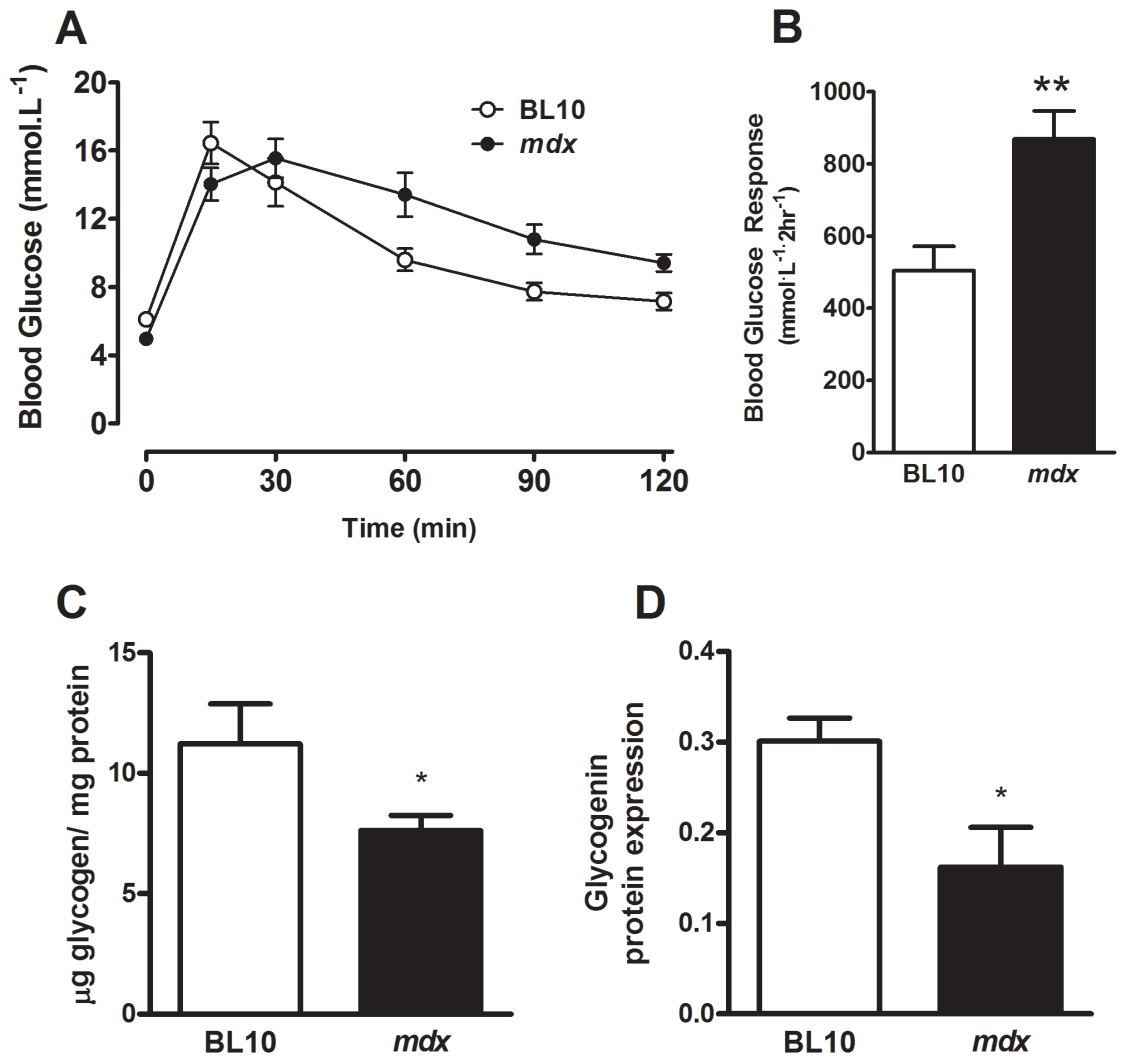
*Mdx* mice are glucose intolerant and have reduced liver glycogen. Means (± SEM) for blood glucose concentration measured at 15, 30, 60, 90 and 120 min after the *i.p.* injection of 1 g/kg glucose in BL10 and *mdx* mice (**A**). Blood glucose response in BL10 and *mdx* mice during the glucose tolerance test (expressed as area under the curve above baseline) (**B**). n = 8; **P<0.01 compared with skeletal muscle from BL/10 wild type mice. Means (± SEM) for liver glycogen concentration (**C**) and glycogenin (**D**) protein expression in fed wild type (BL10) and *mdx* dystrophic mice; n = 8; *P<0.05 compared with skeletal muscles from BL/10 wild type mice.

The increased muscle glycogen in *mdx* mice led us to measure glucose tolerance since altered glycogen metabolism is usually indicative of altered glucose metabolism. As shown in Figure 4A/B, *mdx* mice had an altered blood glucose response during the glucose tolerance test and exhibited a 30% increase in the area under the curve. These observations suggest that liver glycogen metabolism could also be altered similar to that previously reported in animal models of diabetes [23], [24]. Indeed, we found that glycogen content was significantly lower in liver lysates from *mdx* mice compared with control mice as shown by biochemical analysis (Fig. 4C). In addition, glycogenin, the glycogen primer, was measured as described and its expression was decreased significantly (Fig. 4D).

### Decreased Liver Glycogen in mdx Mice is not Caused by Changes to Glycogen Metabolism or Glycogen Particle Structure

**Figure 5:**
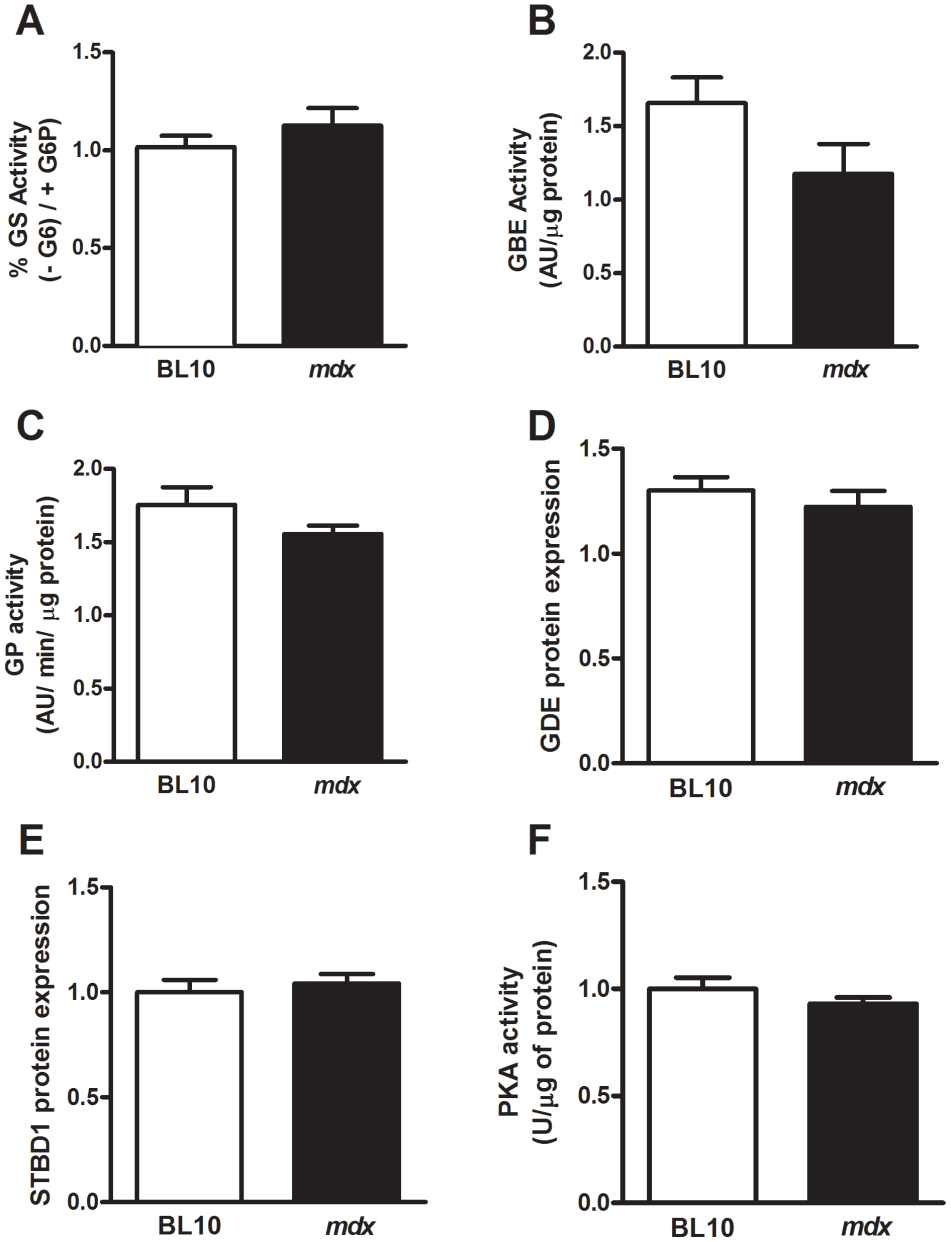
Liver glycogen metabolism is unchanged in *mdx* mice. Means (± SEM) for glycogen synthase (**A**), glycogen branching enzyme (**B**), glycogen phosphorylase (**C**) activity, glycogen debranching enzyme protein expression (**D**), STBD1 protein expression (**E**) and PKA activity (**F**); n = 8. No differences were observed between groups.

To determine whether the observed decrease in liver glycogen content in *mdx* mice could be attributable to changes in addition to glycogenin (Fig. 4D), we investigated enzyme activities and protein expression levels that we found altered in skeletal muscles of *mdx* mice (Fig. 2 and 3). We found no change in GS, GP or GBE activities in liver lysates between wild type and *mdx* mice (Fig. 5A–C). In addition, the protein expression levels of GDE and STBD1 were also unchanged in liver lysates of wild type and *mdx* mice (Fig. 5D–E) suggesting no changes to glycogen particle synthesis or degradation rates. Finally, we measured PKA activity (Fig. 5F) and found no significant changes between liver lysates of *mdx* and wild type mice supporting the data described in Figures 5A and 5C.

## Discussion

Glycogen metabolism is impaired in many diseases associated with hyperglycaemia. Animals with Type 1 diabetes have increased renal glycogen [25] but decreased liver glycogen [24] whereas animals with Type 2 diabetes have increased fasting liver glycogen [26] and increased cardiac muscle glycogen [27]. In this study we report that *mdx* dystrophic mice have impaired glucose tolerance, increased skeletal muscle glycogen and decreased liver glycogen. Enzymes important for skeletal muscle glycogen regulation including glycogenin, GS, GP, GBE and GDE were all significantly different in *mdx* mice compared with controls. The *mdx* mice also had decreased glycogenin expression in the liver. Thus the absence of the structural protein dystrophin in skeletal muscle leads to alterations in glucose metabolism in tissues not restricted to skeletal muscle.

New glycogen particles are initiated by the enzyme glycogenin that covalently attaches a chain of glucose residues to a specific amino acid within its polypeptide chain [20]. Thus, the two-fold increase in glycogenin protein expression in *mdx* mice indicated a two-fold increase in the number of glycogen particles. The new glycogen particle is further elongated by the activities of GS and glycogen branching enzyme. Glycogen synthase activity is increased in skeletal muscles of *mdx* mice at both a protein phosphatase-mediated dephosphorylation (leading to active GS) and allosteric activation by G6P (Fig. 2) suggesting increased phosphorylation by an upstream protein kinase. Interestingly, we found decreased GBE activity in skeletal muscles of *mdx* mice, a phenomenon that has never been demonstrated outside of glycogen storage disease Type III specific for this enzyme [28]. Decreased GBE activity suggests a lower number of branch points and therefore a glycogen particle that would be a poor substrate for GP and so more difficult to degrade when energy was required. A new pathway to manage less branched glycogen has recently been proposed – glycogen-specific autophagy; termed ‘glycophagy’ [21]. A key protein thought to target less branched glycogen for this pathway is called starch-binding domain protein 1 (STBD1) [29]. This protein contains a carbohydrate-binding domain that is essential for glycogen association [29], a transmembrane domain and a sequence that results in binding to the autophagic protein GABARAPL1 [22]. In support of this pathway being activated, we find STBD1 mRNA and protein expression increased in skeletal muscles of *mdx* mice (Fig. 3F). We suggest that STBD1 tethers less branched glycogen to membranes and, by an as yet undefined mechanism involving interaction with GABARAPL1, participates in the trafficking of glycogen to the lysosome; the process of glycophagy. Since STBD1 shows a preference for binding to poorly branched glycogen, it could favor the disposal of aberrant glycogen particles and be part of a quality-control mechanism especially in the absence of GP activity (Fig. 3). Alternatively, it might be controlled by conditions or stimuli that tend to produce incorrectly branched glycogen.

Characterization of the glycogenin promoter has led to the identification of several transcription factor binding sites including a cAMP response element-binding (CREB) site [30]. Indeed in skeletal muscle cells, glycogenin mRNA expression was inhibited with increasing concentrations of cAMP [30]. It has been demonstrated previously that cAMP levels are increased in muscles from *mdx* mice [31], which would support the reduction in glycogenin mRNA expression observed in dystrophic muscle (Fig. 2E). We suggest that glycogenin mRNA expression is negatively regulated in an attempt to reduce the glycogen concentration in muscles of *mdx* mice, by a yet to be identified pathway. Despite this observation, we find that dystrophic muscles had increased glycogenin protein expression (Fig. 2D) and decreased PKA activity (Fig. 3B). These latter two findings suggest that cAMP concentrations are decreased. PKA normally phosphorylates GS leading to reduced activity and phosphorylates GP-kinase that in turn phosphorylates GP leading to increased activity. However, the opposite is found in skeletal muscles of *mdx* mice contributing to the increased glycogen concentration agreeing with a reduced PKA activity. Reduced PKA activity has previously been identified in muscles from *mdx* mice because of its inability to bind to myospryn, an PKA-anchoring protein (AKAP) that associates with the dystrophin-glycoprotein complex [32]. Furthermore, these AKAPs are known to create microdomains of cAMP signaling events that are completely disorganized in dystrophic muscle [33]. Therefore, we suggest that both increased and decreased cAMP downstream signaling events are possible within skeletal muscles of *mdx* mice.

Mild glucose intolerance has been reported previously in DMD patients [34] and more commonly in patients with myotonic dystrophy [35]. Given that hyperglycemia in diabetic mice can lead to alterations in hepatic glycogen [23], [24] we measured the glycogen concentration in liver lysates from wild type and *mdx* mice and found that glycogen was decreased significantly in *mdx* mice; similar to that found in animals with Type I diabetes [24]. Investigation of the enzymes important for glycogen metabolism revealed only reduced glycogenin protein expression in the *mdx* mice. One mechanism for this finding is that circulating interleukin-6 (IL-6) is increased in *mdx* mice compared with wild type mice [36] and this is known to reduce total liver glycogen [37]. Whether IL-6 signaling represses the glycogenin promoter is unknown but worthy of further investigation.

In conclusion, we found that *mdx* mice exhibit several metabolic phenotypes, including hyperglycemia and aberrant glycogen metabolism in both skeletal muscle and the liver. The molecular basis for increased skeletal muscle glycogen is the presence of poorly branched glycogen particles together with decreased PKA activity leading to post-translational dysregulation of both glycogen synthase and glycogen phosphorylase.

## Supporting Information

Figure S1 **Representative western blots** for glycogenin, starch-binding domain protein 1 (STBD1) and glycogen-debranching enzyme (GDE) in skeletal muscle and liver lysates from BL/10 and *mdx* mice. Glyceraldehyde-3-phosphate (GAPDH) was used as loading control.
(PDF)
