# Supplementary material for: Dysfunctional Muscle and Liver Glycogen Metabolism in mdx Dystrophic Mice
Source: PLoS One. 2014 Mar 13;9(3):e91514. doi: 10.1371/journal.pone.0091514 (PMC3953428; doi:10.1371/journal.pone.0091514)
Supplement: Figure S1 — Representative western blots for glycogenin, starch-binding domain protein 1 (STBD1) and glycogen-debranching enzyme (GDE) in skeletal muscle and liver lysates from BL/10 and mdx mice. Glyceraldehyde-3-phosphate (GAPDH) was used as loading control. (PDF) [file pone.0091514.s001.pdf]

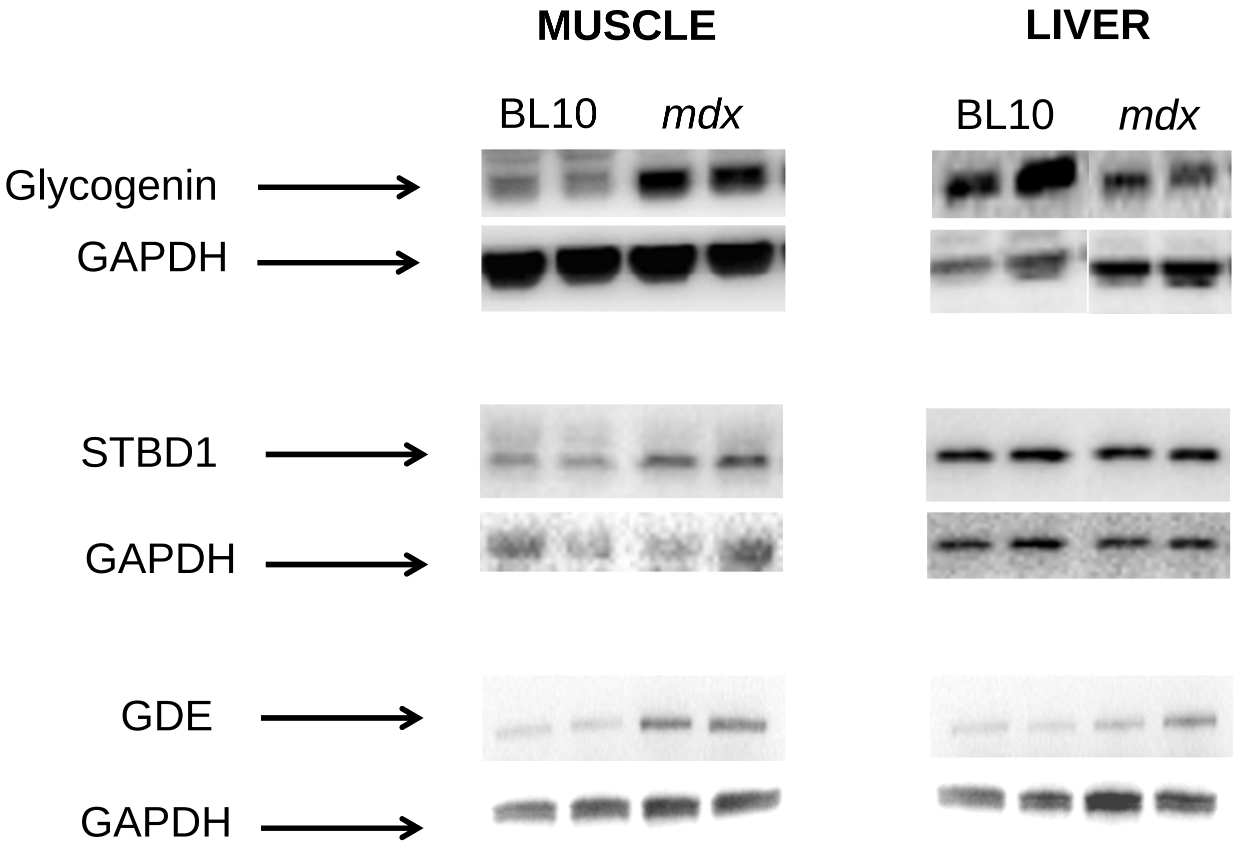

Supplemental figure 1: representative western blots for glycogenin, starch-binding domain protein 1 (STBD1) and glycogen-debranching enzyme (GDE) in skeletal muscle and liver lysates from BL/10 and *mdx* mice. Glyceraldehyde-3-phosphate (GAPDH) was used as loading control.
